# An exploration of symptom burden and its management, in Saudi Arabian patients receiving haemodialysis, and their caregivers: a mixed methods study protocol

**DOI:** 10.1186/s12882-019-1424-9

**Published:** 2019-07-09

**Authors:** Bushra Alshammari, Helen Noble, Helen McAneney, Peter O’Halloran

**Affiliations:** 1 School of Nursing and Midwifery, Queen’s University Belfast, Medical Biology Centre, 6th floor - room 06.313, 97 Lisburn Rd, Belfast, BT9 7BL UK; 2 College of Nursing, University of Hail, P.O. Box 2440, Hail city, Saudi Arabia; 3 Centre for Public Health, Queen’s University Belfast, Institute of Clinical Sciences, Block B, Grosvenor Road, Belfast, BT12 6BJ UK

**Keywords:** Symptom burden, Symptoms, Management, Haemodialysis, Dialysis, End-stage kidney disease, Chronic kidney disease, Caregiver, Burden, Saudi Arabia, Mixed methods research

## Abstract

**Background:**

Globally 10% of the population worldwide are affected by chronic kidney disease (CKD), making it one of the most prevalent chronic diseases. Several studies have highlighted that the symptoms of CKD have a significant impact on patients. A number of symptoms, including fatigue and depression, are associated with poor patient health, increased risk of hospitalisation and mortality. Physical and emotional symptoms often remain under-recognised and largely untreated; however, patients often create a variety of self-management strategies to meet the challenges of these symptoms. There is a lack of knowledge regarding symptom burden and the experiences of patients receiving haemodialysis (HD) and their caregivers, particularly in Saudi Arabia, therefore, this study aims to explore symptom burden and its management amongst patients receiving HD in addition to caregiver burden.

**Method:**

A mixed methods, sequential, explanatory design consisting of two phases: phase 1 involves a cross-sectional study design with a planned convenience sample size of 141 patients who will be recruited from King Khaled hospital, Saudi Arabia. Thirty-two physical and psychological symptoms will be measured using the Chronic Kidney Disease-Symptom Burden Index (CKD-SBI). Additionally, 130 caregivers will complete the Arabic version of the Zarit Burden Interview (ZBI-22) to identify the level of burden in the caregivers of patients on maintenance HD. Phase 2 of the study is a qualitative descriptive design involving semi-structural interviews with 15 eligible patients currently receiving HD. The selection of participants for interviews will be based on the patients’ total CKD-SBI scores with five individuals recruited from the lowest, median and highest percentiles. Additionally, 15 caregivers of the patients to be interviewed, will also be recruited and interviewed.

**Discussion:**

This study focuses on a wide number of physical and psychological symptoms experienced by patients receiving HD. It will also focus on the effective management strategies patients employ to help reduce their perceived symptoms. Burden in caregivers of patients receiving HD will also be explored. Furthermore, the association between symptom burden and caregiver burden will be investigated. Findings from this study will provide evidence to help health care providers to develop effective interventions to assess and manage symptoms in patients receiving HD.

## Background

Chronic kidney disease (CKD) is an escalating global health problem. It is characterized by a gradual decrease in the ability of the kidneys to function effectively over time [1]. According to the National Kidney Foundation [1], 10% of the population worldwide are impacted by CKD, making it one of the most prevalent chronic diseases. Currently, Saudi Arabia and Belgium have the highest estimated CKD prevalence (24%), followed by UK and Singapore (16%). Norway and the Netherlands have the lowest estimates at 5% [2]. The treatment that is most preferred in end-stage renal disease (ESRD) is renal transplantation [3]. However, this treatment is not always possible due to a shortage of donors and appropriate medical facilities [4–6] or the ineligibility of recipients for kidney transplantation due to their health condition [6, 7]. Hence most patients rely on dialysis treatment. Approximately 90% of all patients receiving dialysis are undergoing HD [6]. Although dialysis is life-saving, patients on maintenance HD experience multiple physical and emotional symptoms that impact on their well-being [8]. HD is exhausting for patients who commonly experience a range of symptoms such as fatigue, depression, anxiety, itching, vomiting and nausea [9–11]. Controlling symptoms in patients receiving HD is essential and requires comprehensive prior assessment. However, studies claim that healthcare providers fail to commonly recognise and treat the physical and emotional symptoms experienced by patients receiving HD [12]. In a recent study, the main barriers to symptom management is that health care providers are unaware of patient symptoms [13]. According to Solano [14], patients with ESRD experience a similar degree of symptom distress to cancer patients. Several studies suggest that patients with symptom burden create a self-management strategy to help reduce or, relieve and cope with their chronic disease symptoms [15–17]. The development and use of these self-management strategies requires further exploration and understanding.

A comprehensive literature search identified a significant number of studies which have assessed various physical and psychological symptoms of CKD, in a range of countries, including USA, [18–21] UK, [22, 23], and Canada [24]. A single cross sectional study has assessed the various physical and psychological symptoms of CKD, for individuals living in Saudi Arabia [11]. Studies suggest that demographic data and clinical variables can potentially influence the individual’s symptom experiences in different diseases [11, 18, 20, 22, 23, 25–30]. Previous studies have failed to explore symptom burden, its impact and its management using a mixed methods explanatory study design, particularly in Saudi Arabia.

Patients with CKD often rely on others to help with their medical needs and daily living activities [31]. Family caregivers are essential partners in the delivery of complex healthcare services. The number of people living with patients receiving HD has increased due to the increase in the prevalence of kidney disease [32]. The most frequent responsibilities undertaken by caregivers of those receiving HD, include supervision of the patient’s nutrition and hygiene, driving patients to treatment sessions, and administering medications. Completing these responsibilities may have negative life experience for caregivers of patients receiving HD [33]. Recent literature suggests that the informal caregiver role in patient requiring dialysis, causes feelings of being overwhelmed and greater burden [34]. There is a lack of evidence regarding understanding the association between caregiver burden and patient burden.

There are a number of factors associated with different experiences of caregiver burden such as living with the patient [35], age [36–39], gender [37–43], education [39], socioeconomic status [33, 44], relationship to the patient [37, 38] and comorbidity [41, 45, 46]. The burden on caregivers of chronically ill patients has received less attention and commonly focuses on psychiatric illness such as dementia [47–52], breast cancer [53, 54] and with limited research interest considering caregiver burden in CKD [34, 55, 56].

This study aims to explore symptom burden, its self-management, and the factors predicting symptom burden in patients undergoing HD. It also aims to assess the level of burden in the caregivers of these patients, the relationship between patient and caregiver burden, and to explore factors that may influence reporting of patient and caregiver burden.

## Research questions

This study seeks to answer the following research questions:

Phase 1-A quantitative phase with patients

- What is the level of symptom burden for patients receiving HD?
- What are the factors that predict symptom burden among patients receiving HD?

Phase 1-B Quantitative phase for caregivers.

For caregivers of patients receiving HD:

- What is the level of caregiver burden?
- What are the factors that predict caregiver burden?
- What is the association between patient symptom burden and caregiver burden?

Phase 2 Qualitative phase - patients and caregivers

- What is the experience of symptom burden for patients receiving HD?
- What is the experience of caregiver burden for caregivers of patients receiving HD?
- What management strategies are used by patients receiving HD to manage symptoms?

Integration question:

How do the insights gained from the qualitative data help to explain the impact of symptom burden and the factors associated with symptom and caregiver burden identified in the quantitative analysis?

## Design and methods

**Fig. 1:**
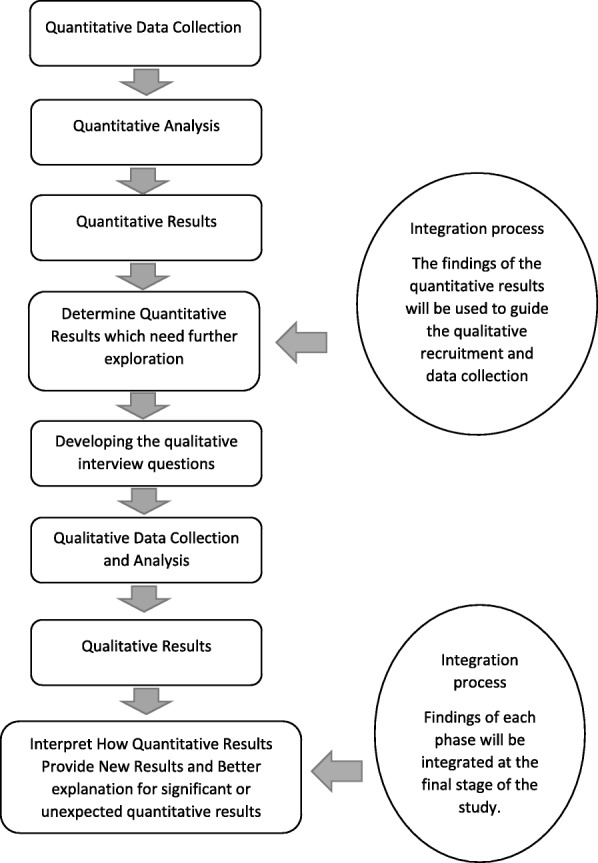
Research process for Explanatory Sequential Design

The study will apply a mixed methods sequential explanatory design with a quantitative phase followed by a qualitative phase [57]. The aim of this study is not only to identify symptom burden, its management, and the factors predicting patient and caregiver burden; it also aims to provide an understanding of the impact of living with burden and how these factors may influence reporting of caregiver and patient burden. Phase 1 will use a cross-sectional design to measure symptom burden using the *Chronic Kidney Disease Symptom Burden Index (CKD-SBI)* Arabic version questionnaire [58]. Caregiver burden will be collected using the 22-item version of the *Zarit Burden Interview* (ZBI-22) [59]. Phase 2 is a qualitative descriptive design involving a number of semi-structured interviews, which will aid the interpretation of Phase 1 findings, by providing a context within which the quantitative data can be understood [57]. A sequential explanatory design can be used to enhance understanding of quantitative research findings by providing supporting evidence from the qualitative phase of the study [60]. The main focus of qualitative interviews is to provide an in-depth understanding of the significant, unexpected or unexplained results which may arise during the quantitative phase, such as individuals who reported extreme symptom burden scores, either low or high [61, 62]. Qualitative interviews will also explore the impact of living with symptoms in patients receiving HD and their caregivers as well as to discuss the self-management strategies that might be used by patients to reduce or relieve their symptoms. In this study, the quantitative phase will be the dominant area of investigation; with the qualitative phase taking a secondary explanatory role. The quantitative data will be analysed and these provisional results will be used to help guide qualitative data collection. When both qualitative and quantitative data is collected, results will be integrated to provide insight into symptom experience, caregiver experience and how predictors contribute to increase the level of burden. It will also help to illustrate any differences between the management strategies used by patients with different levels of symptom burden. (see Fig. 1).

## Setting

The research will be completed within the HD centre at King Khaled Hospital, Hail City, in northern Saudi Arabia. This 284 bed hospital contains various specialist areas, including the HD centre. Approximately 240 patients attend the HD centre each week. The centre is open 6 days a week; each day is divided into two shifts (morning and evening). Each shift receives around 60 patients for HD treatment. The King Khaled Hospital is the only hospital within the region that provides HD treatment for patients with ESRD.

## Study population

### Patients

To be eligible for study recruitment patients must have received regular HD therapy for more than 3 months, be able to communicate in English or Arabic, are aged 18 years or older, and are cognitively able to participate in the study. Cognitive assessment will be completed by the nephrologist within the HD treatment area. Patients receiving peritoneal dialysis or conservative management will be excluded from participation in the study.

### Caregivers

Eligible caregivers must be aged over 18 years and be identified by the patient as the key caregiver who provides some level of practical help and support for more than 3 months. Caregivers will only be recruited into the study when patients consent to their participation. They must be able to communicate, read and write in English or Arabic. Caregivers who do not meet the *eligibility* criteria will be excluded.

## Participants

### Sample size

The primary outcome of this study is to measure symptom burden in patients receiving HD. The study will also identify the impact on symptom burden in relation to the following possible predictors: age, gender, education level, marital status, income, co-morbidity, employment, living distance from hospital, and the duration of dialysis. A power calculation was performed using GPower software [63] to help avoid type 11 error and calculate the sample size required. Given a medium effect size (f^2^ = 0.15), significance level of 5, 90% power and 9 predictors, the sample size required is 141 patients receiving HD. Similarly, for 7 predictor variables, the study needs to recruit 130 caregivers. The number of people available in the hospital who receive HD is 240. A previous study involving this population was able to recruit a larger number of participants [58], so this study is likely to recruit the required number of participants to help ensure the credibility of findings.

For the exploratory qualitative interviews, we anticipate a sample of approximately 15–20, participants in both patient and caregiver groups will be sufficient to achieve data saturation [64]. The data emerging from respondent interviews will be kept under review as interviews progress, and no further interviews will be completed when it becomes clear that no new themes will emerge from subsequent data [65, 66]. Decisions can therefore be made prior to coding and thematic development [67].

## Recruitment (see Fig. 2)

### Phase 1: cross-sectional study

A renal unit staff member, will act as a gatekeeper and will be involved in the process of pre-screening and recruiting patients at the centre. If potential participants who meet the inclusion and exclusion criteria acknowledge interest, the gatekeeper will introduce them to the researcher. Participants will be provided with an information sheet outlining the study and a consent form, to be returned if they are willing to participate. Participants will be given at least 24 h to consider whether they wish to participate in the study or not. When patients sign the consent form, the questionnaire will be distributed and completed by patients during their HD sessions. The researcher will provide explanations and assistance to the patients who are unable to complete the questionnaire, due to either a lack of understanding or physical restrictions imposed by the dialysis treatment, such as the presence of cannulas or cuff pressure on the patients’ arms.

**Fig. 2:**
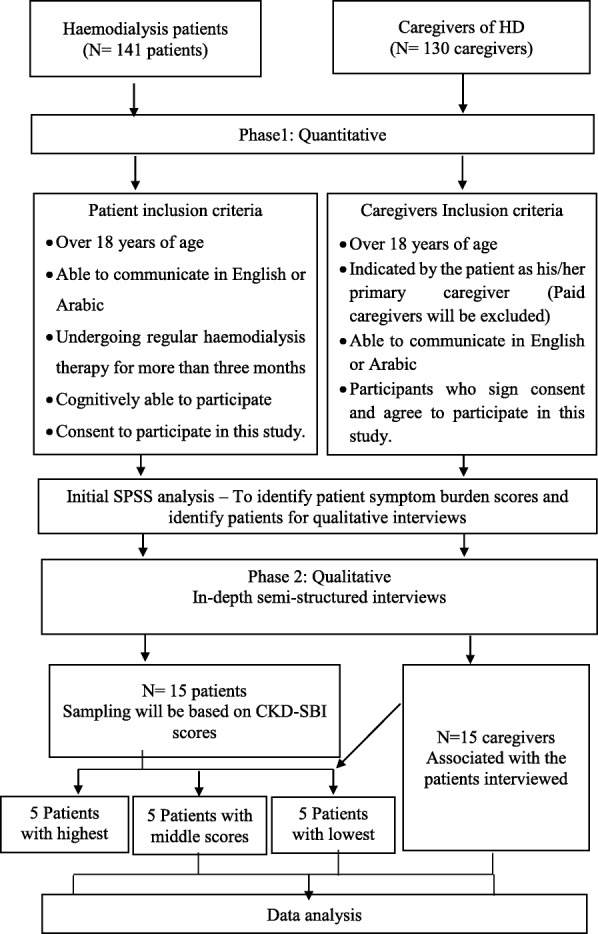
Flow diagram illustrating study procedures

Patients will be asked to identify the person who is primarily involved in their care, this will allow identification of the most appropriate caregivers to be included in the study. The information sheet and caregiver questionnaire will then be sent to caregivers in a sealed envelope via the patient. The information sheet will include an invitation to contact the researcher should the caregiver have any questions. The sealed envelope will also include a separate form asking caregivers whether they are happy to be contacted at a later date to determine if they would like to be interviewed (consent to be contacted form). Questionnaires, and (if the caregiver wishes, the consent to be contacted form) will be returned to the researcher in the stamped addressed envelope provided. Returning the questionnaire will be judged to imply consent.

The literature reports that the majority of caregivers are likely to be women [68], and that female patients report higher symptom burdens [11]. We will collect data on the proportion of participating patients and caregivers who are women and this will be taken into account in our regression analysis.

### Phase 2: qualitative descriptive design

The researcher will identify those patients with the top five highest, median and lowest scores from the CKD-SBI using SPSS and their caregivers. If these patients and their associated caregivers refuse to participate in the study, participants with the next highest, median and low scores will be recruited. Potential participants will be contacted and provided with an information sheet explaining the nature and purpose of interviews and consent. The researcher and study participants will meet at an agreed private location to conduct each interview. Caregivers who prefer a telephone interview will be sent a consent form, which can be returned via the patient or by the supplied and stamped addressed envelope. Once consent is obtained the telephone interview will be arranged. To help maximise the response rate, caregivers who do not return the questionnaire within a three-week period will be sent a reminder letter via the patients. The interview questions will be formulated following the analysis of quantitative findings. Interviews will be of approximately 30–60 min duration.

## Instruments

### Patients

Symptom burden will be measured using the Chronic Kidney Disease Symptom Burden Index (CKD-SBI) Arabic version questionnaire [11]. CKD-SBI is the modified version of the Dialysis Symptom Index (DSI) and used widely to assess symptoms in CKD and ESRD. Testing of the Arabic CKD-SBI has shown it to have good psychometrics and excellent internal consistency (Cronbach’s α = 0.91) as well as good reliability and validity in CKD population [58]. The CKD-SBI was adapted to assess various aspects of symptom burden [11]. These include assessment of the burden of the disease, and the prevalence, frequency, distress and severity of each symptom. A total of 32 CKD symptoms are assessed. Three empty fields were provided to allow individuals to add symptoms not identified on the symptom list. The purpose of the prevalence scale is to assess each symptom’s presence or absence using (Yes/No). Prevalence scores range from 0 to 32. 0 is lowest prevalence score while a score of 32 is the highest possible score. Three further factors will be measured (distress, severity and frequency) on a scale of 0 to 10 for each respondent’s rating and will be recorded as, Distress: from none to highly distressed; Severity: none to extremely intense; and Frequency: from never to frequent. The highest possible score for each scale is 320, which is considered to constitute extremely high burden.

### Caregivers

The ZBI scale was developed to assess the level of burden for those caring for patients with dementia. It has recently been used to assess caregiver burden in CKD [55, 69, 70]. It contains 22 items, which examine five caregiver burden domains: burden on the relationship; loss of control over life; finance; social and family life; and emotional well-being. The questionnaire items will be rated using a five-point Likert scale, with 0 (rarely) being the lowest, and 4 (nearly always) being the highest. Zarit et al. [49] suggest summing all response scores to show the level of caregiver burden, with 0 to 20 showing no burden or slight burden; 21 to 40 indicating mild to moderate burden; 41 to 60 moderate and severe burden; and 61 to 88 indicating a heavy burden [71]. ZBI was translated into Arabic and shows good validity [72, 73]. Reliability of the translated instruments was also tested and found to be 0.97 [48]. The ZBI has excellent internal consistency α = 0.83 [74], and α = 0.89 [49, 75]. User agreement required to use the Arabic version of ZBI was obtained from the Mapi Research Trust.

## Data sources (method of assessment)

### Phase 1: cross-sectional study

The primary outcome of this study is to explore symptom burden and its management in patients receiving HD using the CKD-SBI. In addition, demographic information will be collected and will include: age; gender; marital status; education level; employment status; ethnicity; income, distance from hospital. Co-morbidities and duration of dialysis will be obtained from dialysis charts or hospital records. Co-morbidity will be measured using the Davies Co-morbidity Index [76]. To measure caregiver burden, ZBI-22 will be used. The demograph characteristic of caregivers will be also obtained. All instruments that will be used in the study have demonstrated validity and reliability [58, 59].

### Phase 2: qualitative descriptive design

In-depth semi-structured qualitative interviews will be completed to identify the management behaviours used by patients receiving HD to reduce their symptom distress. Semi-structured interviews will be used to encourage, respondents to discuss important matters that may have been missed during the quantitative stage of the study. Participants will have the opportunity to voice their personal, unique ways of managing, controlling and coping with symptoms. Each interview will also explore their experiences of renal and non-renal health care and other support services. The impact of burden in both patients’ and caregivers’ lives also will be investigated. Caregivers will be interviewed separately, to ensure that the information they provide will not be influenced by the presence of patients. This will help to ensure the accuracy of information, and assist in obtaining a deeper understanding of the caregiving experience, identifying the negative and positive effects of providing care.

## Data analysis

### Phase 1: cross-sectional study

Simple descriptive statistics analysis will be provided for the scale and categorical variables using the Statistical Package for the Social Sciences (SPSS). For scale variables, mean and standard deviation will be reported for the normally distributed data, or alternatively, median and interquartile range (IQR) will be used for the skewed distributed data. For categorical variables, absolute frequency (n) and relative frequency (percentage) (%) of the response, such as gender, education level, marital status, monthly income, and HB level will be reported. Correlations between any pairs of continuous variables will be presented using Pearson’s correlation coefficients. Chi-square or Fisher’s exact tests for categorical data and continuous variables will be used to highlight associations between both samples. Multiple linear regression analysis will be used for both samples to identify any association between burden scores, demographics characteristics and other variables.

### **Phase 2:** qualitative descriptive design

Interviews will be analysed using thematic analysis [77, 78]. Audio-recorded interviews will be transcribed in Arabic by the researcher and translated by a certified bilingual translator into English. The researcher will review the final version of the translation to ensure the credibility of translation. The translator will sign a non-disclosure form to guarantee confidentiality of the participants’ data. The data will then be stored electronically to allow for coding and analysis. Coding will be managed using NVivo qualitative data analysis software Version 10 [79]. The analysis will be based on three phases: data reduction, data display and conclusion drawing/verification process [66]. Findings will be discussed and verified with researcher colleagues at every stage to ensure the accuracy of interpretation, ensure reliability and promote rigour. By the use of coding researchers can identify themes and patterns in interviews’, this can be determined by the words used including word frequency, relationships between words, and how communication is structured [80]. Codes can then be displayed or organised to give greater clarification of the content and allow the drawing of conclusions.

## Integration

This is the process of mixing, linking or interacting between the qualitative and quantitative findings of a study. This process is a significant element of mixed methods research [81]. There are several approaches to integrating qualitative and quantitative research methods and data [60, 82]. In this study, integration will occur at the design, methods and interpretation stages of the study.

Integration through design: an exploratory sequential mixed methods design using both qualitative and quantitative research approaches is suitable to gain a comprehensive understanding of the symptom experience of patients receiving HD, and to help understand the factors which may increase the level of symptom burden. It is also important to understand the variety of symptom management strategies which are used by patients with high, median and low levels of symptom burden.

Integration at the methods stage, occurs by linking the method of data collection or analysis [83]. In this study, two approaches will be used to ensure linking, including connection and building [84]. Connection will be achieved when participants are selected from the population who respond to the survey. Building occurs when quantitative research findings are used to inform sample selection for the qualitative phase of the study. Based on symptom burden scores, in the quantitative phase of the study, patients will be selected to participate in semi-structured interviews. Patients with high, middle and low symptom burden scores, will be assigned to be involved in semi-structured interviews. Building will also be achieved when results from quantitative data analysis, inform qualitative data collection. Interview questions will be modified based on the quantitative study findings to help meet the aim of the study..

Integration at the interpretation stage will be achieved using a narrative approach that describes the quantitative and qualitative results thematically [84]. Quantitative and qualitative findings will be synthesized through narratives, in the study results and discussion by the use of weaving, where the data weaves back and forth, around similar themes or concepts [84]. This approach will help to provide comparisons and contrasts between findings and help to draw out new insights beyond the symptom experience, identified in the quantitative and qualitative findings.

## Rigour

In a mixed methods design, researchers need to ensure the validity and trustworthiness of the study findings. The following strategies will be applied to enhance rigour in the sequential explanatory design: the use of well-validated and reliable instruments to collect data in the dominant quantitative phase, determination of the important findings that emerge from the quantitative phase to help recruit participants and guide data collection in the qualitative exploration, analysing the qualitative data independently and then in relation to the quantitative findings. Findings will be discussed and verified with the involved researchers at every stage to assess the accuracy of the interpretation, improve reliability and ensure a rigour. To ensure credibility and transferability, thick descriptions will be provided to enable judgments about how the results are believable and reflected in the data and how likely the context of this study will fit with other populations, settings and contexts. The Good Reporting of A Mixed Methods Study (GRAMMS) guidelines will be used to assist the quality of reporting and help enhance the transparency of the study processes [85].

## Study approvals

The study protocol has been approved by the Research Ethics Committees at Queen’s University Belfast, UK, in September 2017, reference number 10.BAlshammari05.17.M6.V1. It has also been approved by Hail University, Saudi Arabia, and the research and education centre of King Khaled Hospital, Saudi Arabia, where the study will be conducted. The study is sponsored by the Ministry of Education, University of Hail, Saudi Arabia and permission to access patients at the HD centre has been granted.

## Discussion

This study will use a sequential explanatory mixed methods design to explore a wide number of symptoms and explain symptoms experienced by patients receiving HD. It will also coneptualises the management strategies patients use to help cope with these symptoms. The research findings will make a significant contribution to understand the symptoms experienced in patients receiving HD. This is the first study which utilizes a qualitative approach to explore the symptom experience and caregiver burden in patients receiving HD in Saudi Arabia. In-depth understanding of symptom burden will provide guidance for health care providers to develop interventions for assessing and managing symptoms in the future. Appropriate interventions to manage symptoms will help to improve the quality of care and lead to improved health-related quality of life for patients with CKD. This study also provides valuable insights into caregivers experiences of patients receiving HD which will enable health professionals to better understanding caregiver burden and related stress. Finally, the collection of both qualitative and quantitative data will facilitate a more holistic understanding of symptom burden and management in both patients and their caregivers.

The limitations of this study include the use of a convenience sample in the quantitative phase of the study. With the use of convenience sampling there is an increased risk of bias, as study participants may not accurately reflect the characteristics of the total population. An additional limitation of the study is that interviews will be translated from Arabic to English. It is possible that the real meanings of Arabic words may be misinterpreted with translation. To help address this potential limitation, the quality of translations will be assessed independently by an additional bilingual translator to ensure accuracy.

## Data Availability

Not applicable.

## Abbreviation

CKD: Chronic kidney disease
CKD-SBI: Chronic Kidney Disease Symptom Burden Index
ESRD: End-stage renal disease
HD: Haemodialysis
ZBI-22: The 22-item version of the Zarit Burden Interview
